# Editorial: 50 years of BMT: conditioning regimens and early complications after transplantation

**DOI:** 10.3389/fonc.2024.1369573

**Published:** 2024-02-07

**Authors:** Michele Malagola, Raffaella Greco, Jean El Cheikh

**Affiliations:** ^1^ Unit of Blood Diseases and Stem Cell Transplantation, Azienda Socio Sanitaria Territoriale (ASST)-Spedali Civili of Brescia, Department of Clinical and Experimental Sciences, University of Brescia, Brescia, Italy; ^2^ Unit of Hematology and Bone Marrow Transplantation, Istituto di Ricovero e Cura a Carattere Scientifico (IRCCS) San Raffaele Hospital, Vita-Salute San Raffaele University, Milano, Italy; ^3^ Division of Hematology/Oncology, Department of Internal Medicine, American University of Beirut Medical Center, Beirut, Lebanon

**Keywords:** allogeneic stem cell transplantation, conditioning, graft versus host disease, infections, conditioning intensity

Since 1957, when the first Allogeneic Stem Cell Transplantation (Allo-SCT) was performed by E. Donnall Thomas, thousands of transplants have been registered worldwide, in particular for acute myeloid leukemia (AML). Indications continue to rise across haematological diseases, solid tumours and immune disorders. More than six decades later, the most significant evolutions of allo-SCT regard: i) the spectrum of diseases that are nowadays curable with this procedure (now including malignant and non-malignant conditions) and ii) the upper limit of age for allo-SCT eligibility (now approaching 75-77 years) (1). Other most notable developments include the success of unrelated donor and haploidentical SCT, an increase followed by decrease in the number of cord blood transplants. These aspects, next to the massive expansion of SCT technology, gradually shed a light on the issue of conditioning platforms and of transplant-related mortality (TRM), particularly in the first 3-6 months after allo-SCT.

The Research Topic entitled “ *50 Years of BMT: Conditioning Regimens and Early Complications After Transplantation* “ collected 4 Manuscripts.

Yanada et al. reported a very interesting analysis of the real-world data on conditioning regimens for AML in Japan. Over more than 20 years of activity, more than 1,000 myeloablative (MAC) and more than 4,000 reduced intensity conditioning regimens (RIC) have been registered. Total body irradiation (TBI) was the backbone of the great majority of MAC, whereas Busulfan and Melphalan were equally distributed among the RIC regimens. The MAC-RIC ratio mildly decreased between 2000 and 2005, but then it remained stable over time, with more than 60% of the transplants performed with high-intensity conditioning.

Liu et al. reported on a retrospective series of 608 consecutive patients with SAA, divided into those with criteria for SAA at diagnosis, those who progressed from non-SAA to SAA (both groups treated with immunosuppression) and those who progressed from non-SAA to SAA, and were treated with allo-SCT. The most important evidence is that immunosuppression resulted less effective in SAA progressing from non-SAA, but allo-SCT can improve the long-term outcomes.

In another Manuscript of the Research Topic reported, Colita et al. analysed autologous transplants (ASCT) for lymphomas on behalf of the Romanian Society for Bone Marrow Transplantation. In particular, they focused on 222 ASCT performed with three types of conditioning: BEAM, CLV (cyclophosphamide, lomustine, etoposide) and LEAM (lomustine, etoposide, cytarabine and melphalan). The overall response rate at day 100 was comparable across the three regimens (ranging from 70 to 80%). The relapse-free survival at 2 years was significantly better for BEAM regimen, followed by LEAM and CLV, but this did not translate into a survival benefit. Transplant-related mortality was similar across the three regimens, and the most frequent grade 3/4 non-hematologic toxicity was mucositis, with a higher incidence in the BEAM group. Finally, Zhou et al. described a case report focusing on the role of metagenomic next-generation sequencing (mNGS) in the diagnosis and treatment of disseminated visceral Kaposi sarcoma after allo-SCT. Through whole-exome sequencing, germline mutations in the FANCI and RAD51 were detected: these genes are associated with impairment of DNA repair, leading to tumor susceptibility. The research of these germline mutations in donor’s DNA may be of help for the complete understanding of the mechanisms underlying the onset of secondary neoplasms after allo-SCT.

The issue of conditioning regimens is still a major point in the field of allo-SCT. Myeloablation and stem cells’ engraftment was originally obtained with Total Body Irradiation (TBI) alone or in combination with cyclophosphamide. Subsequently, TBI was gradually replaced by alkylators, namely busulfan, and from the ‘80s several different conditioning regimens have been developed. These regimens have been historically classified into three main categories: myeloablative (MAC), non-myeloablative (NMA) and reduced intensity (RIC). This classification, based on the alkylators’ dose and on the role of myeloablation versus immunosuppression in each transplant platform, has been widely used in the last 20 years, both in retrospective and prospective studies. In more recent years, a new category of conditionings has been proposed: the reduced toxicity conditioning regimens, such as Fludarabine and Busulfan for 4 days – FB4, that showed fully myeloablative effects, but with reduced extra-hematological toxicity. Following the evolution of the transplant platform, including new drugs (e.g. Treosulfan or Clofarabine) (2, 3) and new Graft Versus Host Disease (GVHD) prophylaxis (e.g. post-transplant cyclophosphamide) a new score for conditioning intensity has been proposed by the Acute Leukemia Working Party of the European group for Blood and Marrow Transplantation (ALWP-EBMT) (4). This Transplant Conditioning Intensity (TCI) score seems to be highly reproducible and categorizes the different conditionings into 3 categories (low-intermediate-high), with a clear impact on TRM and relapse incidence. Among the new drugs proposed for conditioning in adult patients, particularly with AML or myelodysplastic syndromes (MDS), Treosulfan is surely one of the most attractive one. The combination of Fludarabine and Treosulfan 10 g/sqm/day for 3 days (FT10) has become highly recommended for elderly patients with AML or MDS, particularly with advanced-phase disease, following the international registrative trial (2). Moreover, Treosulfan has been used in combination with a second alkylator (e.g. Melphalan) in the setting of haploidentical SCT (5). Finally, the retrospective data from our Gruppo Italiano Trapianto di Midollo (GITMO) showed that Treosulfan has been used in Italy for older AML/MDS patients, with high-risk diseases, but this did not translate into an impairment of long-term outcomes with respect to Busulfan-based conditioning regimens (6). All these data suggests that this drug has highly *in vivo* anti-leukemic activity. The issue of dose is still a matter of debate, considering that it can be used at a dose up to 14 g/sqm/day for 3 days (FT14). This latter dose is currently under evaluation in an Italian prospective phase II trial.

Moving to the topic of early complication after allo-SCT, in particular during first 3-6 months after transplant, two major aspects should be considered: i) infectious complications and ii) acute GHVD. One of the most impressive improvement of infections’ prevention in the transplant setting has been represented by the introduction of Letermovir (LET) prophylaxis against CMV from day 0 to day +100 after transplant. LET was proved to significantly reduce CMV clinically significant infections (CMV-csi) and CMV disease, in the context of a multicentric, international, randomized trial. The results of the registrative study have been confirmed in several single and multicentric experiences and recent metanalysis grouped these data together, showing that LET significantly reduces both CMV-related events, but also mortality for all causes, NRM and, in some cases, significantly improved Graft and Relapse Free Survival (GRFS) (7). The extension of LET prophylaxis in high-risk patients beyond day +100 (until day +200) confirmed a reduction in the incidence of CMV-csi with respect to placebo (8). Although CMV has significantly reduced its impact in patients submitted to allo-SCT, other issues, such as the role of anti CMV specific immune reconstitution, are currently under study (9).

Moving to acute GVHD, we can assess that, in the last 10 years, its incidence has maintained within 30-40% (considering grade II to grade IV cases). This result surely must be improved, but it should be considered within the evolution of the transplant scenario worldwide. In fact, it is undoubtedly that an increase in patients’ age and in the spectrum of diseases addressed to allo-SCT (e.g. myeloproliferative neoplasms) has been registered in many Countries. These two aspects are well known risk factors for acute GVHD, and the stability in the incidence of this complication in the last 10 years is probably the effect of an amelioration of the supportive care and a reduction in extra-hematological toxicity (e.g. gastrointestinal toxicity). Moreover, the availability of new drugs such as Ruxolitinib significantly changed the prognosis of steroid-refractory acute GVHD and contributed to the reduction of TRM. This latter aspect, together with the improvement in relapse prevention with the introduction of post-SCT maintenance (e.g. hypo-methylating agents, midostaurin,….), is associated with a progressive amelioration of overall survival and relapse free survival after allo-SCT in the most recent years (10).

In conclusion, we think that allo-SCT is now entering a very challenging era, with the advent of other cellular therapies (e.g. CART cells therapies) and with the evolution of other immune therapies (e.g. new molecular target drugs, monoclonal antibodies, bi-specific antibodies). In this context, allo-SCT should be integrated in the therapeutic program of each disease (in particular AML). Prospective and controlled trials are strongly warranted in order to improve anti-leukemic activity and reduce toxicity, both in terms of infections and in terms of GVHD, maintaining high attention to the interplay between all these factors (**Figure 1**).

**Figure 1:**
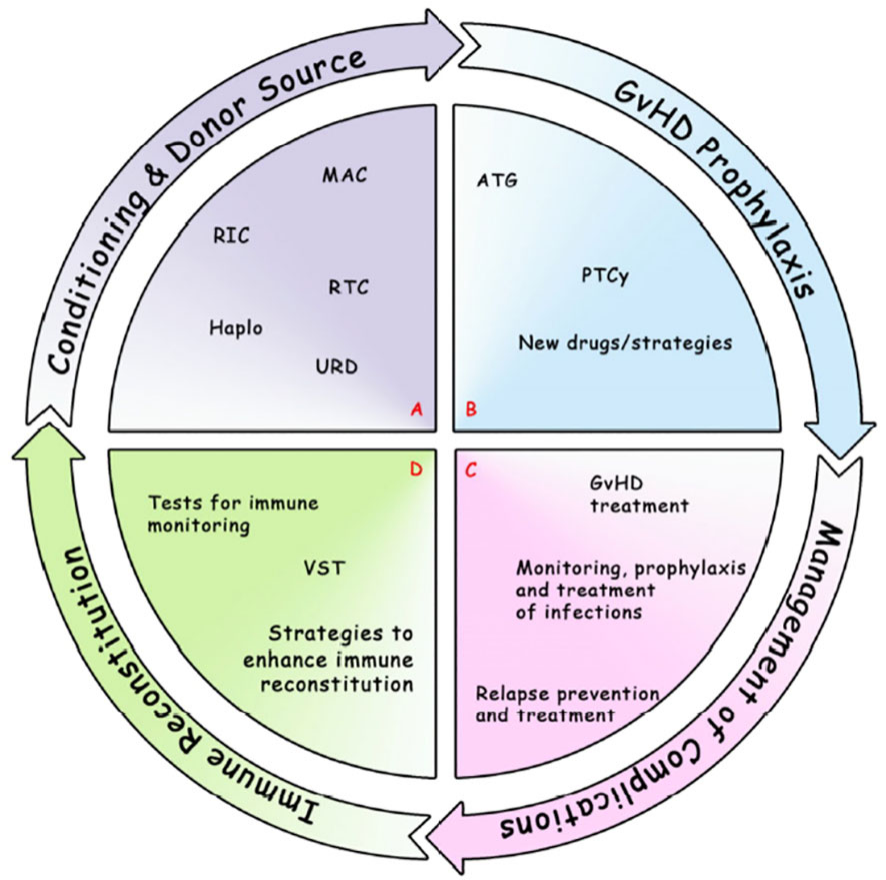
Most notable and recent developments in allo-SCT field. **(A)** Conditioning regimen and donor source, including MAC, RIC, RTC, Haplo, URD. **(B)** GvHD prophylaxis, including ATG, PTCy, new drugs/strategies. **(C)** Management of complications: GvHD treatment (eg. IST, ECP, mAb, TKIs, JAKi, FMT, MSC); relapse prevention and treatment (maintenance, eg. DLI, multikinase inhibitor, small molecules, HMA, CART); monitoring, prophylaxis and treatment of infections (eg. bacteria, fungi, CMV, EBV, VZV, HHV6, SARS-CoV-2). **(D)** Immune Reconstitution: tests for immune monitoring (eg. routine tests [absolute lymphocyte counts, lymphocyte subsets, antibody titers], assays currently performed in the research setting [eg. functional assays, viral-specific response, measures of thymic output and T/B cell repertoire]; VST; strategies to enhance immune reconstitution (eg. Treg-based therapies, graft manipulation). allo-SCT, allogeneic Stem Cell Transplantation; ATG, anti-thymocyte globulin; CART, chimeric antigen receptor; CMV, Cytomegalovirus; DLI, donor lymphocyte infusion; EBV, Epstein Barr Virus; ECP, extracorporeal photopheresis; FMT, fecal microbiota transplantation; GvHD, graft-versus-host disease; Haplo, haploidentical; HHV6, Human Herpes Virus 6; HMA, hypomethylating agents; IST, immunosuppressive agents; JAKi, Janus kinase inhibitors; mAb, monoclonal antibody; MAC, myeloablative conditioning; MSC, mesenchymal stromal cells; PTCy, post-transplant cyclophosphamide; RIC, reduced intensity conditioning; RTC, reduced toxicity conditioning; SARS-CoV-2, severe acute respiratory syndrome related coronavirus 2; TKIs, Tyrosine kinase inhibitors; Treg, T regulatory cells; URD, unrelated donor; VST, viral-specific T cells; VZV, Varicella-Zoster Virus.

## Author contributions

MM: Conceptualization, Data curation, Formal analysis, Funding acquisition, Investigation, Methodology, Project administration, Resources, Software, Supervision, Validation, Visualization, Writing – original draft, Writing – review & editing. RG: Conceptualization, Data curation, Formal analysis, Funding acquisition, Investigation, Methodology, Project administration, Resources, Software, Supervision, Validation, Visualization, Writing – original draft, Writing – review & editing. JE: Conceptualization, Data curation, Formal analysis, Funding acquisition, Investigation, Methodology, Project administration, Resources, Software, Supervision, Validation, Visualization, Writing – original draft, Writing – review & editing.
